# Neutralizing Antibody Activity Against the B.1.617.2 (delta) Variant Before and After a Third BNT162b2 Vaccine Dose in Hemodialysis Patients

**DOI:** 10.3389/fimmu.2022.840136

**Published:** 2022-03-04

**Authors:** Louise Benning, Katrin Klein, Christian Morath, Marie Bartenschlager, Heeyoung Kim, Mirabel Buylaert, Marvin Reineke, Maximilian Töllner, Christian Nusshag, Florian Kälble, Paula Reichel, Paul Schnitzler, Martin Zeier, Caner Süsal, Ralf Bartenschlager, Matthias Schaier, Claudius Speer

**Affiliations:** ^1^Department of Nephrology, University of Heidelberg, Heidelberg, Germany; ^2^Department of Infectious Diseases, Molecular Virology, University of Heidelberg, Heidelberg, Germany; ^3^Department of Virology, University of Heidelberg, Heidelberg, Germany; ^4^Institute of Immunology, University of Heidelberg, Heidelberg, Germany; ^5^Transplant Immunology Research Center of Excellence, Koç University Hospital, Istanbul, Turkey; ^6^German Center for Infection Research, Partner Site Heidelberg, Heidelberg, Germany; ^7^Division Virus-Associated Carcinogenesis, German Cancer Research Center, Heidelberg, Germany; ^8^Department of Molecular Medicine Partnership Unit Heidelberg, European Molecular Biology Laboratory, Heidelberg, Germany

**Keywords:** SARS-CoV-2, COVID-19, hemodialysis, variants of concern, delta variant

## Abstract

Hemodialysis patients are at high risk for severe COVID-19, and impaired seroconversion rates have been demonstrated after COVID-19 vaccination. Humoral immunity wanes over time and variants of concern with immune escape are posing an increasing threat. Little is known about protection against the B.1.617.2 (delta) variant of concern in hemodialysis patients before and after third vaccination. We determined anti-S1 IgG, surrogate neutralizing, and IgG antibodies against different SARS-CoV-2 epitopes in 84 hemodialysis patients directly before and three weeks after a third vaccine dose with BNT162b2. Third vaccination was performed after a median (IQR) of 119 (109–165) days after second vaccination. In addition, neutralizing activity against the B.1.617.2 (delta) variant was assessed in 31 seroconverted hemodialysis patients before and after third vaccination. Triple seropositivity for anti-S1 IgG, surrogate neutralizing, and anti-RBD antibodies increased from 31/84 (37%) dialysis patients after second to 80/84 (95%) after third vaccination. Neutralizing activity against the B.1.617.2 (delta) variant was significantly higher after third vaccination with a median (IQR) ID_50_ of 1:320 (1:160–1:1280) compared with 1:20 (0–1:40) before a third vaccine dose (*P*<0.001). The anti-S1 IgG index showed the strongest correlation with the ID_50_ against the B.1.617.2 (delta) variant determined by live virus neutralization (r=0.91). We demonstrate low neutralizing activity against the B.1.617.2 (delta) variant in dialysis patients four months after standard two-dose vaccination but a substantial increase after a third vaccine dose. Booster vaccination(s) should be considered earlier than 6 months after the second vaccine dose in immunocompromised individuals.

## Introduction

The current coronavirus disease 2019 (COVID-19) pandemic has led to more than 270 million cases and around 5.3 million deaths worldwide as of December 2021 (1). COVID-19 vaccination has been proven safe and effective in preventing severe COVID-19 disease with more than 8 billion vaccine doses already administered globally (1). However, hemodialysis patients are still at great risk for severe COVID-19 because of advanced age, underlying comorbidities, and premature aging of the immune system resulting in lower humoral and cellular vaccine response (2, 3).

Impaired seroconversion rates after two-dose BNT162b2 vaccination have been shown in hemodialysis patients with seroconversion rates in the range of 71-96% (4, 5). Recently, first real-world data investigated the effectiveness of mRNA vaccination in 12,169 hemodialysis patients: vaccinated hemodialysis patients had a lower risk of COVID-19 infection as well as a significantly lower incidence of hospitalization or death compared with unvaccinated patients (6). However, waning humoral immunity has been demonstrated in healthy and dialysis populations as early as three months after second vaccine dose, leading to an increase in breakthrough-infections (7–9).

Emerging variants of concern (VoCs) such as B.1.1.7 (alpha), B.1.351 (beta), and B.1.617.2 (delta) with partial immune escape are posing an increasing challenge to our health care systems. We and others have demonstrated that hemodialysis patients are not adequately protected against the VoCs B.1.1.7 (alpha) and B.1.351 (beta) after standard two-dose BNT162b2 mRNA vaccination despite detectable seroconversion in commercially available assays testing for anti-wild type antibodies (10, 11). Due to the high risk for severe COVID-19 courses, impaired seroconversion rates after two-dose vaccination, and waning humoral immunity over time, a third vaccine dose has recently been recommended for hemodialysis patients. First results indicate an enhancement of humoral response and seroconversion to positivity in previous non-responders after a third vaccine dose (12). However, little is known about neutralization against the B.1.617.2 (delta) variant in hemodialysis patients before and after third vaccination. Only recently, Liu et al. showed a modest reduction for BNT162b2-elicited neutralization against the B.1.617.2 (delta) variant compared to the parental pandemic strain in healthy volunteers (13).

Characterizing humoral responses to vaccination is necessary to estimate possible protection from severe COVID-19 infection and to facilitate clinical decision-making regarding additional booster vaccinations especially for vulnerable cohorts such as hemodialysis patients. The SARS-CoV-2 spike protein was early identified as a major antigenic target for the development of COVID-19 vaccines (14). Antibodies that bind to the spike protein, especially to its receptor-binding domain (RBD), prevent viral attachment to the host cell and neutralize the virus (14, 15). Most serological assays used to determine response to vaccination measure anti-spike IgG or surrogate neutralizing antibodies and are easily applicable in clinical routine (16). However, the gold standard to assess neutralization of SARS-CoV-2 and vaccine-induced cross-neutralization of VoCs are neutralization assays that involve live SARS-CoV-2 viruses produced in cell culture, requiring a Biosafety Level 3 (BSL3) facility (16).

In this study, we characterized humoral responses before and after a third mRNA vaccine dose in 84 hemodialysis patients using a chemiluminescent immunoassay, a surrogate virus neutralization test and a bead-based assay. All three assays are commercially available and feasible in clinical routine. We further assessed vaccine-induced cross-neutralization of the B.1.617.2 (delta) variant using a live-virus assay and compared results to those obtained by commercially available assays.

## Methods

### Study Design

In this prospective, dual-center, observational cohort study, we enrolled 84 hemodialysis patients after two-dose BNT162b2 mRNA vaccination before a third BNT162b2 mRNA vaccine dose between August and September 2021. Serum was collected directly before third vaccination and after a median (IQR) of 21 (20–23) days after third vaccination. The third vaccine dose was administered a median of 119 (109–165) days after the second vaccine dose. To detect patients with active SARS-CoV-2 infection, rapid antigen and PCR testing was performed once a week. Patients with antibodies against the nucleocapsid protein were excluded from the study because of suspected prior SARS-CoV-2 infection.

We determined anti-SARS-CoV-2 spike S1 IgG and neutralizing surrogate antibodies (snAB) in all 84 hemodialysis patients before and after third vaccination. In addition, antibodies against various SARS-CoV-2 target epitopes, including anti-RBD antibodies and anti-spike S1 antibodies against 4 common cold coronaviruses, were assessed. In 31 individuals with detectable anti-S1 IgG, snAB, and anti-RBD seropositivity, neutralizing antibodies present in serum before a third vaccine dose were quantified by using a live virus neutralization assay with the B.1.617.2 (delta) variant. In the same 31 subjects, changes in neutralization against the B.1.617.2 (delta) variant were assessed using serum taken after third vaccination.

The study was approved by the ethics committee of the University of Heidelberg and conducted in accordance with the Declaration of Helsinki. Written informed consent was obtained from all study participants. The study is part of an ongoing single-center study to determine immunogenicity of COVID-19 vaccines in different study populations (DRKS00024632).

### Anti-SARS-CoV-2 Spike S1 IgG and Nucleocapsid Antibodies

Anti-spike S1 IgG antibodies were measured using the SARS-CoV-2 Total Assay (Siemens, Eschborn, German). Results of this assay are obtained in as little as 10 minutes on the Atellica IM Analyzer with a capacity to process up to 440 assays per hour, dependent on test mix. Positivity is defined as a semi-quantitative index of ≥1, which gives a specificity of 100% and a sensitivity of 98% for the detection of anti-S1 IgG antibodies. The assay is called semi-quantitative as there is no scale basis for antibody testing for this assay. The clinical applicability of a semi-quantitative assay is currently unknown and cannot be transferred into a degree of immunity. A strong correlation for anti-S1 IgG as measured by the SARS-CoV-2 Total Assay to neutralization against SARS-CoV-2 wild-type or other SARS-CoV-2 variants of concern has been described previously (10, 17–19). We further used the Elecsys anti-SARS-CoV-2 assay (Roche, Mannheim, Germany) to detect antibodies against the nucleocapsid protein. Assays were performed according to the manufacturers’ instructions.

### SARS-CoV-2 Specific Surrogate Neutralizing Antibodies

A surrogate virus neutralization test (Medac, Wedel, Germany) was applied to detect snAB in a sample, as described previously by us and others (5, 7, 10, 17–23). Samples were pre-incubated with horseradish peroxidase (HRP) conjugated recombinant SARS-CoV-2 RBD fragment (HRP-RBD). This allows binding of the circulating neutralizing antibodies to the HRP-RBD. The pre-incubated samples are then added to a capture plate which is pre-coated with human ACE2 receptor protein (hACE2). Unbound HRP-RBD and any HRP-RBD with unspecific binding is captured on the plate whereas HRP-RBD bound to neutralizing antibodies remains in the supernatant and is removed during washing. Optical density at 450 nm was measured in each well and the percent (%) inhibition was calculated as follows:


$$=\left(1-\left(\frac{\text{OD value of Sample}}{\text{OD value of Negative Control}}\right)\right)\times 100\%$$

With a cut-off of ≥30% inhibition of RBD : ACE-2 binding, the test achieves 99.9% specificity with 95-100% sensitivity to detect surrogate neutralizing antibodies (20). Testing for antibodies against the RBD region of the SARS-CoV-2 spike protein gives additional information regarding neutralizing capacity of antibodies as many antibodies raised against RBD have neutralizing potential. Antibodies against the RBD have shown to be the major source of SARS-CoV-2 neutralizing antibodies, but additional non-RBD antibodies are also known to neutralize SARS-CoV-2 (16, 24–26). Therefore, single-antigen neutralization assays do not fully reflect the total pool of neutralizing antibodies (16).

### IgG Antibodies Against Various SARS-CoV-2 Epitopes and 4 Common Cold Coronaviruses

To identify IgG antibodies against different SARS-CoV-2 epitopes and the spike protein of 4 other common cold coronaviruses, a multiplex bead-based assay for the Luminex platform (LabScreen COVID Plus, One Lambda Inc., West Hill, CA, USA) was performed (27). The assay simultaneously detects antibodies against 4 distinct fragments of the SARS-CoV-2 spike protein, namely the full spike protein, the spike S1, the spike S2, and the RBD of the spike protein and thus results in a broader characterization of humoral responses after infection or vaccination. This assay is especially applicable in HLA/transplant laboratories that already use the Luminex platform to detect and identify HLA antibodies in transplant candidates (27).

In addition, antibody reactivity against the spike S1 protein of 4 other common cold coronaviruses, namely HCoV-229E, HCoV-HKU1, HCoV-NL63, and HCoV-OC43 was measured to rule out possible cross-reactivity. We assessed the mean fluorescence intensity (MFI) using a Luminex 200 device (Luminex Corporation, Noord-Brabant, The Netherlands). For each target, individual cut-off values are given by the manufacturer (**Supplementary Table S1**).

### Cross-Neutralization Against the B.1.617.2 (delta) Variant of Concern

Neutralization titers were determined in titration experiments on VeroE6 cells as described previously by us (10, 28). SARS-CoV-2 virus stocks were produced by amplification of the B1.617.2 (delta) strain isolated from nasopharyngeal and oropharyngeal swabs of PCR-confirmed SARS-CoV-2-positive patients in VeroE6 cells, as previously described by us and others (10, 17–19, 23, 29). Stocks were stored at –80°C until use. For neutralization experiments, two-fold serial dilutions of sera were incubated with B.1.617.2 (delta). After 1h at 37°C, the mixture was added to VeroE6 cells and cells were fixed in the plates with 5% formaldehyde 24h later. Virus replication was determined by immunostaining for the viral nucleocapsid protein using an in-cell ELISA. Values were normalized to those obtained with cells infected in the absence of patient serum (100% infection) and non-infected cells (0% infection), the latter determining the assay background. The ID_50_ equates the serum dilution that reduces infection of cells by 50%. The cut-off for detection of this neutralization assay is at a neutralization titer of 1:10.

### Assessing Reactogenicity

Any adverse events were assessed using a 12-item questionnaire as previously described (**Supplementary Methods**) (21, 22). We inquired common local and systemic reactions such as pain at the injection site, redness, swelling, fever, chills, fatigue, headache, muscle ache, joint pain, and the use of medication one week after first, second, and third vaccination, respectively.

### Statistics

Data are given as median and interquartile range (IQR) or number (N) and percent (%). The Mann-Whitney *U* test was applied for statistical analysis of continuous variables. In paired analysis of antibody levels, the Wilcoxon rank-sum test was used. To describe the relationship between anti-S1 IgG, surrogate neutralizing, and anti-RBD anti-wild-type antibodies to vaccine-induced cross-neutralization against the B.1.617.2 (delta) variant as determined by a live virus assay, we calculated Spearman’s rho as a nonparametric measure of rank correlation. Statistical significance was assumed at a *P*-value <0.05. The statistical analysis was performed using GraphPad Prism version 9.0.0 (GraphPad Software, San Diego CA, USA).

## Results

### Study Population

We prospectively enrolled 84 hemodialysis patients with two-dose BNT162b2 mRNA vaccination directly before a third vaccine dose. Median (IQR) age of hemodialysis patients was 72 (62–79) years, and 26 (31%) participants were females. Dialysis vintage was a median of 46.3 (14.8–89.3) months. Seven (8%) hemodialysis patients were previously transplanted and 16 (19%) on low-dose immunosuppressive maintenance therapy due to previous transplantation or autoimmune disease. Baseline characteristics are given in **Table 1**.

**Table 1:** Baseline characteristics.

Number of patients, N	84
Age at enrollment (years), median (IQR)	72 (62–79)
Sex (female), N (%)	26 (31)
BMI, median (IQR)	25.1 (21.9–28.3)
Dialysis vintage (months), median (IQR)	46.3 (14.8–89.3)
Kt/V, median (IQR)	1.6 (1.4–1.8)
Cause of nephropathy	
Vascular, N (%)	7 (8)
Diabetes, N (%)	18 (21)
Glomerular disease, N (%)	28 (33)
PKD, N (%)	10 (12)
Cardiorenal, N (%)	6 (7)
Systemic, N (%)	3 (4)
Other, N (%)	12 (14)
Comorbidities	
Arterial hypertension, N (%)	79 (94)
Diabetes, N (%)	34 (41)
Cancer, N (%)	21 (25)
CAD, N (%)	46 (55)
PAD, N (%)	25 (30)
Chronic lung disease, N (%)	19 (22)
Chronic liver disease, N (%)	12 (14)
Previously transplanted, N (%)	7 (8)
Immunosuppressive medication, N (%)	16 (19)

BMI, body mass index; CAD, coronary artery disease; N, number; PAD, peripheral artery disease, PKD, polycystic kidney disease.

### Humoral Responses Before and After a Third Dose of BNT162b2 mRNA Vaccination

**Figure 1:**
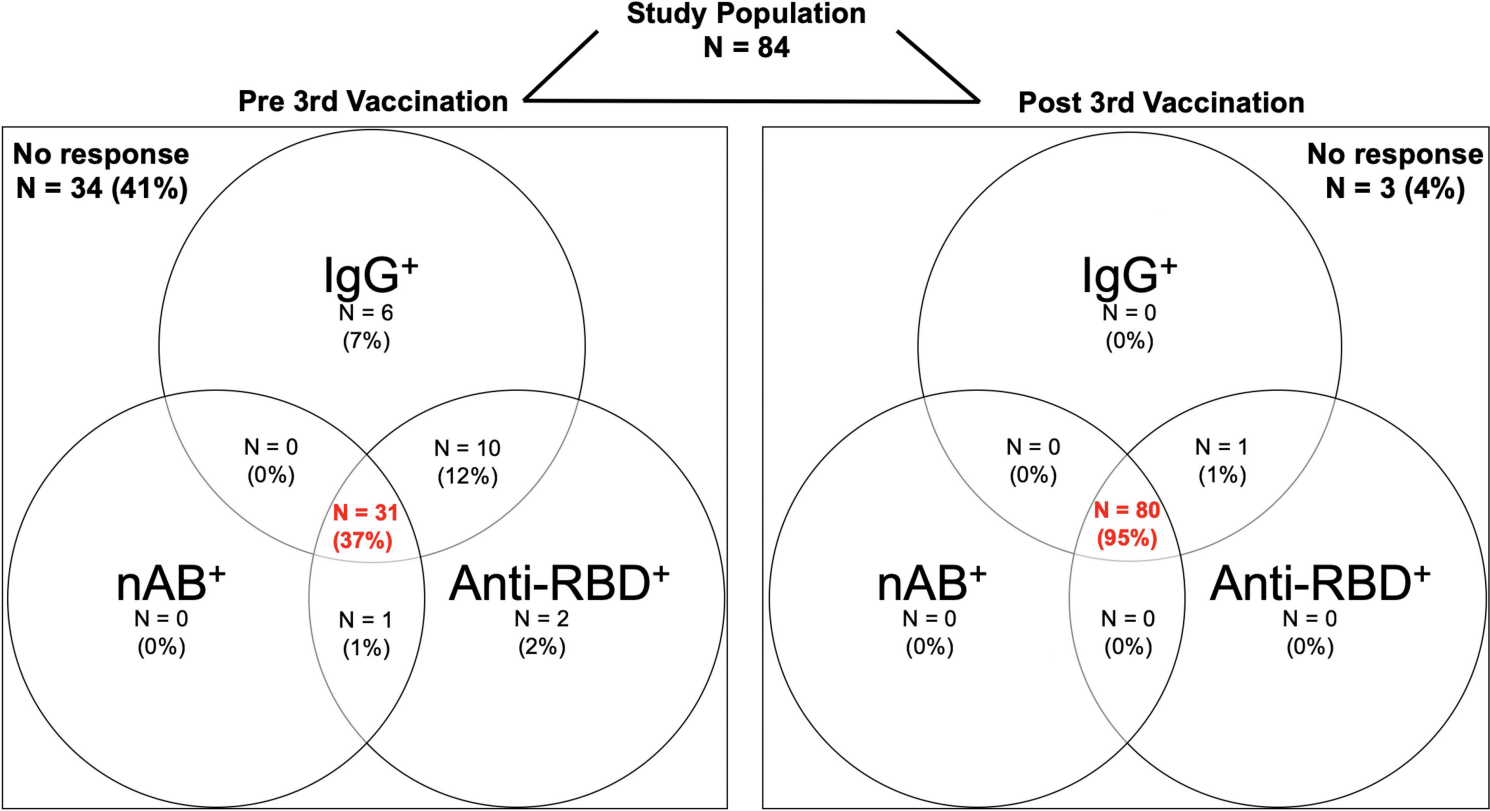
Study population for assessment of humoral response before and after a third BNT162b2 vaccine dose in hemodialysis patients. Seropositivity for anti-S1 IgG, surrogate neutralizing and anti-receptor-binding domain (RBD) antibodies before and after a third BNT162b2 vaccine dose in 84 hemodialysis patients shown in a Venn-Diagram. Seropositivity was defined as an anti-S1 IgG index ≥1 in a chemiluminescent immunoassay, an inhibition ≥30% in a surrogate virus neutralization test, and a mean fluorescence intensity (MFI) ≥5800 in a bead-based multiplex assay. The red, bolt numbers in the middle of each panel indicate the proportion of patients with seropositivity for all three assays. N, number; nAB, neutralizing antibodies; RBD, receptor-binding domain.

Before the third vaccine dose, 34/84 (41%) hemodialysis patients did not show seroconversion in any of the three commercially available assays with either an anti-S1 IgG index ≥1, surrogate neutralizing antibodies with an inhibition of ≥30%, or anti-RBD antibodies with an MFI ≥5800 (**Figure 1**). After third vaccination, only 3/84 (4%) hemodialysis patients had no detectable antibodies in any of the three assays. In contrast, the proportion of hemodialysis patients with seropositivity in all three assays increased from 31/84 (37%) to 80/84 (95%) after third vaccine dose.

**Figure 2:**
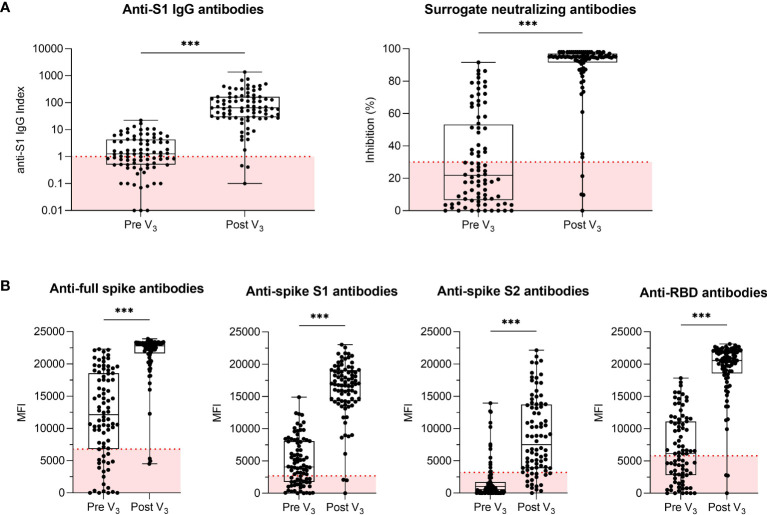
Humoral response in hemodialysis patients before and after a third BNT162b2 vaccine dose. **(A)** Anti-S1 IgG and surrogate neutralizing antibodies in 84 hemodialysis patients before and after a third BNT162b2 vaccine dose. An anti-S1 IgG index ≥1 and an inhibition ≥30% as indicated by the dashed red line defined seroconversion for anti-S1 IgG and surrogate neutralizing antibodies, respectively. **(B)** IgG antibodies against SARS-CoV-2 full spike, spike S1, spike S2 and receptor-binding domain (RBD) as determined by a bead-based multiplex assay. The y-axis represents the mean fluorescence intensity (MFI) with a red dashed line indicating the cut-off for each respective target. Anti-RBD antibodies were chosen to define the seroconverted cohort for later live virus neutralization as the RBD of the SARS-CoV-2 spike protein is a major target of neutralizing antibodies that block viral attachment to the host cell. MFI, mean fluorescence intensity; RBD, receptor-binding domain; V, vaccination; ***P < 0.001.

Anti-S1 IgG index and surrogate neutralizing antibodies increased from a median (IQR) index of 1.3 (0.5–4.3) to 65 (28.9–165.0) and a median (IQR) % inhibition of 22 (7–53) to 95 (92–97), respectively (for both *P*<0.001; **Figure 2A**). IgG antibodies against the full spike, the spike S1, the spike S2, and the spike RBD also increased significantly after third vaccination from a median (IQR) MFI of 12,116 (6,806–18,546) to 22,774 (21,640–23,172), 4,075 (1,761–8,075) to 16,953 (14,279–19,070), 551 (14–1,722) to 7,515 (3,865–13,726), and from 6,130 (2,829–11,109) to 20,551 (18,558–21,920), respectively (for all *P*<0.001, **Figure 2B**). When analyzing antibody levels in hemodialysis patients with respect to current immunosuppressive therapy, little to no significant differences in antibody levels were found (*P*<0.05 for anti-spike S1; non-significant for anti-S1 IgG, snAB, anti-full spike IgG, anti-spike S2 IgG, and anti-spike RBD; **Supplementary Figure S1**). No significant differences in antibodies against the spike S1 protein of 4 common cold coronaviruses were detected in hemodialysis patients before and after third vaccination (**Supplementary Figure S2**).

### Vaccine-Induced Cross-Neutralizing Antibody Activity Against the B.1.617.2 (Delta) Variant in Seroconverted Hemodialysis Patients

**Figure 3:**
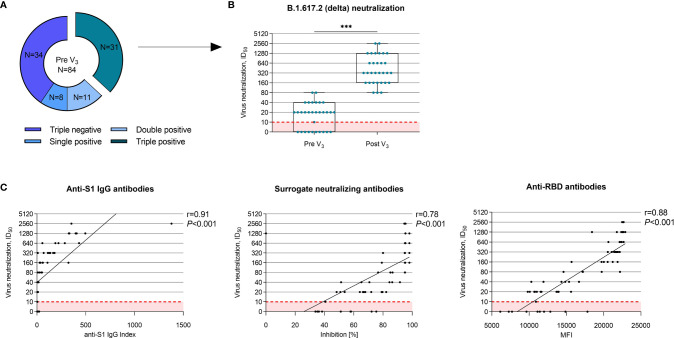
Vaccine-induced cross-neutralization against the B.1.617.2 (delta) variant of concern in seroconverted hemodialysis patients. **(A)** Prior to a third vaccine dose, 31/84 (37%) hemodialysis patients showed a seroconversion in all three commercially available assays, namely anti-S1 IgG ≥1, surrogate neutralizing antibodies with an inhibition ≥30% and anti-RBD antibodies with an MFI ≥5800. In these 31 individuals, neutralization against the B.1.617.2 (delta) variant was performed using a live virus assay. **(B)** Neutralization against the B.1.617.2 (delta) variant before and after a third vaccine dose in 31 hemodialysis patients. The ID_50_ indicates the serum dilution that inhibits 50% of the infectivity. A neutralization titer of 1:10 is the cut-off for this test, indicated by a dashed red line. **(C)** Correlation of commercially available tests such as anti-S1 IgG, surrogate neutralizing and anti-RBD anti-wild-type antibodies to vaccine-induced cross-neutralization against the B.1.617.2 (delta) variant. Spearman’s rho as a nonparametric measure of rank correlation was calculated for all assays. ID_50_; inhibitory dilution 50; MFI, mean fluorescence intensity; N, number; V, vaccination; ***P < 0.001.

Vaccine-induced cross-neutralization against the B.1.617.2 (delta) variant using a live virus assay was determined in 31 hemodialysis patients with detectable anti-S1 IgG, surrogate neutralizing, and anti-RBD antibodies prior to third vaccination (**Figure 3A**). Neutralizing activity against the B.1.617.2 (delta) variant increased significantly with a third vaccine dose from a median (IQR) ID_50_ of 1:20 (0–1:40) to 1:320 (1:160–1:1280) (*P*<0.001; **Figure 3B**). The ID_50_ is defined as the inhibitory dilution that results in 50% reduction of normalized signal in diluted sera. Before a third vaccine dose, 10/31 (32%) hemodialysis patients had no detectable neutralization against the B.1.617.2 (delta) variant despite detectable seroconversion for anti-S1 IgG, surrogate neutralizing antibodies, and anti-RBD antibodies. In contrast, after third vaccination, all 31/31 (100%) hemodialysis patients had detectable neutralizing activity against the B.1.617.2 (delta) variant. Individual courses of anti-S1 IgG, surrogate neutralizing antibodies, anti-RBD antibodies, and antibodies against the full spike, spike S1, and spike S2 are shown in **Supplementary Figure S3**.

Commercially available assays for anti-S1 IgG index, surrogate neutralizing antibodies, and anti-RBD anti-wild-type antibodies showed a strong correlation with the ID_50_ against the B.1.617.2 (delta) variant as determined by a live virus assay (**Figure 3C**). The anti-S1 IgG index correlated best to the ID_50_ against the B.1.617.2 (delta) variant with a Spearman’s rho of r=0.91 (**Figure 3C**).

### Reactogenicity After First, Second and Third Vaccine Dose

Local and systemic reactions were assessed using a 12-item questionnaire. Any side effect was noted in 17/73 (23%), 10/45 (22%), and 30/84 (36%) patients after first, second, or third vaccination, respectively. Local reactions such as pain at the injection site, redness, or swelling were the most common reactions with 13/73 (18%), 6/45 (13%), and 20/84 (24%) reporting any of these local events after first, second, or third vaccine dose, respectively (**Supplementary Figure S4**). Fatigue was the most common systemic event with 4/73 (5%), 3/45 (7%), and 14/84 (17%) reporting fatigue after first, second, and third vaccination, respectively. Other systemic events such as fever, chills, headache, or muscle ache were reported in less than 5% after each vaccine dose.

## Discussion

VoCs such as the B.1.617.2 (delta) variant with partial immune escape increasingly lead to breakthrough infections as vaccine- or infection-induced antibodies wane over time. In particular, immunocompromised individuals with impaired vaccination response after two-dose vaccination are at great risk for (severe) COVID-19 infection (30). Therefore, vulnerable cohorts such as hemodialysis patients were prioritized for a third mRNA vaccine dose. To guide further vaccination strategies, data on immunogenicity after a third vaccine dose, including data on neutralization against the B.1.617.2 (delta) variant, are greatly needed.

We demonstrate a significant increase for each, anti-S1 IgG, surrogate neutralizing, and 4 different SARS-CoV-2 anti-spike antibodies in hemodialysis patients with the administration of a third vaccine dose. Only recently, Bensouna et al. and Ducloux et al. showed a significant increase in anti-S1 IgG antibody levels with seroconversion for almost all dialysis patients after a third BNT162b2 vaccine dose, which is in line with our results (12, 31). Dekervel et al. further showed that in more than half of the non-responders after two-dose vaccination, a third mRNA vaccine dose triggered seroconversion (32). In another study by Stervbo et al., cellular immunity also improved significantly after a third vaccine dose in 23 hemodialysis patients with BNT162b2 or mRNA-1273 vaccination (33). Importantly, we did not observe any severe adverse events after vaccination in our study cohort. Only a small proportion of patients reported any systemic reaction to vaccination, with fatigue being the most reported symptom (17% after a third vaccine dose). This is in line with results obtained by Bensouna et al. who showed that vaccination was overall well tolerated in their cohort of dialysis patients (31). Together, these studies indicate that a third vaccine dose is well tolerated in hemodialysis patients and greatly enhances humoral and cellular responses.

In addition, our current study shows that a third vaccine dose significantly boosts the vaccine-induced cross-neutralizing antibody activity against the B.1.617.2 (delta) variant. Before third vaccination, almost one third of hemodialysis patients with detectable anti-wild-type antibodies in commercially available assays did not show any neutralizing activity against the B.1.617.2 (delta) variant. After a third vaccine dose, neutralization against the B.1.617.2 (delta) variant significantly improved in all patients. Our data therefore stress the importance of a third vaccine dose in hemodialysis patients to better protect these vulnerable patients from infection with immune-escaping variants.

Humoral responses to SARS-CoV-2 vaccination in our patient cohort did not differ significantly between hemodialysis patients with current immunosuppressive therapy to those without. The missing significance in seroconversion rates when comparing hemodialysis patients with and without immunosuppressive maintenance therapy may be fully explained by the fact that the immunosuppressed patients in our cohort received only low-dose immunosuppression. It has been demonstrated that seroconversion rates after COVID-19 vaccination are higher for hemodialysis patients when compared to solid organ transplant recipients (34, 35). In solid organ transplant recipients, immunosuppressive drug number and type are major risk factors of seroconversion failure (34). As a significant proportion of solid organ transplant recipients still has impaired seroconversion rates even after a third mRNA vaccine dose, individualized vaccine protocols seem necessary for these vulnerable patients and vaccination prior to transplantation appears mandatory (18, 35–42).

We did not observe any significant differences when comparing antibody levels before and after a third vaccine dose of 4 common cold coronaviruses, namely HCoV-229E, HCoV-HKU1, HCoV-NL63, and HCoV-OC43. These results imply that the vaccine-induced antibodies against the full spike protein and 3 individual domains of the spike protein (S1, S2 and RBD) determined by a bead-based assay using the Luminex platform are SARS-CoV-2 specific. This has been previously demonstrated by Bray et al. that validated the assay using 96 pre-pandemic sera and 42 PCR-confirmed COVID-19 convalescent sera (27). Further, COVID-19 vaccine response does not seem to be influenced by pre-existing immunity against other common coronaviruses. In line with our hypothesis, Loos et al. only recently found that common viral infections including infections with a common coronavirus (HKU1 or NL63), do not influence the functional evolution of SARS-CoV-2 immunity and therefore should not impact diagnostics or shape vaccine-induced immunity (30).

The main limitation of our study is the lack of data on cellular immunity. Therefore, we cannot fully characterize the complexity of vaccine-induced immunity and the possible protection from severe disease in our patients. Despite impaired seroconversion and low humoral response, patients may still be protected from severe disease by cellular immune responses. However, Stervbo et al. recently found a clear association between cellular and humoral immunity after a third mRNA vaccine dose in hemodialysis patients with a strong increase in spike protein-reactive CD4+ T cells after the third dose (33).

Another limitation is the lack of data on specific antibody responses for other immunoglobulin isotypes. As SARS-CoV-2 primarily infects cells at mucosal surfaces, secretory IgA found at mucosal surfaces may play a key role in protecting against the initial viral spread and transmissibility from the mucosa (43, 44). Recently, the importance of IgA in neutralization of SARS-CoV-2 has been described in patients with COVID-19 infection (43, 44). Sterlin et al. found that IgA antibodies dominated the early SARS-CoV-2-specific antibody response, and that serum IgA was more potent than IgG in neutralizing SARS-CoV-2. Wang et al. similarly found that serum IgA responses to SARS-CoV-2 correlated to IgG responses, and that the dimeric, secretory form of IgA found in mucosa is an even more potent neutralizer of SARS-CoV-2 than monomeric IgA or IgG. In a study comparing humoral responses in COVID-19 infected, single-dose vaccinated and two-dose BNT162b2 vaccinated individuals, Tarkowski et al. found primarily IgG-associated antibody responses against SARS-CoV-2 antigens with varying timing and extent after vaccination or COVID-19 infection (45). Anti-RBD and anti-S1 IgA were also significantly elevated in participants after BNT162b2 vaccination, but patterns were more variable than IgG patterns (45). Different studies suggest the development of vaccines that target a potent specific respiratory IgA response to SARS-CoV-2 (14, 43, 44, 46). In particular, mucosally delivered vaccines that typically produce more robust mucosal immune responses may be of interest in further vaccine development (14, 43, 44, 46).

Further, it remains difficult to deduce neutralization titers that protect from COVID-19 infection or severe disease as no cut-off values that confer protective immunity against severe COVID-19 have been established yet. Khoury et al. approached this issue by modelling protection from SARS-CoV-2 infection across several vaccine and convalescent studies (47). With their modelling data, they estimated *in-vitro* neutralization titers against the wild-type virus between 1:10 and 1:30 that best predicted protection from severe COVID-19 (47). As reduced neutralization titers have been reported for different viral variants, especially immune-escaping variants such as the B.1.351 (beta) variant, vaccine efficacy against these variants is presumably lower (47). However, these correlates of protection were derived from general populations and may not be applicable to immunocompromised cohorts. Therefore, follow-up data on breakthrough infections in hemodialysis patients despite two-dose or even three-dose vaccination with corresponding antibody levels is greatly needed to approach the question of correlates of protection for this vulnerable cohort.

In conclusion, our study indicates that hemodialysis patients are not adequately protected against the B.1.617.2 (delta) variant after standard two-dose mRNA vaccination. A third (booster) vaccination is therefore urgently needed to improve protection against VoCs with partial immune escape. These booster vaccination(s) should be considered earlier than the currently recommended 6 months after the second dose in high-risk individuals. Further studies are needed to assess the longevity of humoral responses acquired with a third vaccine dose and to investigate whether antibodies decline as rapidly as seen after two doses.

## Data Availability Statement

The raw data supporting the conclusions of this article will be made available by the authors, without undue reservation.

## Ethics Statement

The studies involving human participants were reviewed and approved by Ethics committee of the University of Heidelberg. The patients/participants provided their written informed consent to participate in this study.

## Author Contributions

LB and CSp analyzed and interpreted the data and drafted the manuscript. LB, KK, MB, HK, MBu, MR, MT, CN, FK, PR, MS, and CSp collected and managed the data. LB, PS, CSü, and CSp performed experiments on humoral response. MB, HK, and RB performed experiments on live virus neutralization. KK, CM, PS, MZ, CSü, and RB supervised the project and revised the manuscript. All the authors critically reviewed the manuscript. All authors contributed to the article and approved the submitted version.

## Funding

Funding for this study has been received by the Dietmar Hopp Stiftung (grant number: 1DH2111111). LB is funded by the Rahel Goitein-Strauss Program of the Heidelberg Faculty of Medicine. RB is supported by the program for surveillance and control of SARS-CoV-2 mutations of the State of Baden-Württemberg, the German Federal Research Network Applied Surveillance and Testing (BFAST) within the Network University Medicine, the DKFZ@fightCOVID initiative and the Helmholtz Association’s Initiative and Networking Fund Project “Virological and immunological determinants of COVID-19 pathogenesis – lessons to get prepared for future pandemics (KA1-Co-02 “CoViPa”)”. CSp is funded by the Physician Scientist Program of the Heidelberg Faculty of Medicine.

## Conflict of Interest

The authors declare that the research was conducted in the absence of any commercial or financial relationships that could be construed as a potential conflict of interest.

## Publisher’s Note

All claims expressed in this article are solely those of the authors and do not necessarily represent those of their affiliated organizations, or those of the publisher, the editors and the reviewers. Any product that may be evaluated in this article, or claim that may be made by its manufacturer, is not guaranteed or endorsed by the publisher.
