# Supplementary material for: Neutralizing Antibody Activity Against the B.1.617.2 (delta) Variant Before and After a Third BNT162b2 Vaccine Dose in Hemodialysis Patients
Source: Front Immunol. 2022 Mar 4;13:840136. doi: 10.3389/fimmu.2022.840136 (PMC8931261; doi:10.3389/fimmu.2022.840136)
Supplement: Supplementary file 1 [file DataSheet_1.docx]

Supplementary Material

# Table of Contents

Supplementary Table S1 Cut-off values for different SARS-CoV-2 IgG antibodies and antibodies against four common cold coronaviruses.

Supplementary Figure S1 Humoral response in hemodialysis patients before and after a third BNT162b2 vaccine dose with respect to current immunosuppressive therapy.

Supplementary Figure S2 IgG antibodies against four common cold coronaviruses in 84 hemodialysis patients before and after third BNT162b2 vaccination.

Supplementary Figure S3 Individual courses of humoral response in 31 seroconverted hemodialysis patients before and after third BNT162b2 vaccination.

Supplementary Figure S4 Radial graph for reactogenicity in hemodialysis patients after first, second and third BNT162b2 vaccine dose.

Supplementary Methods

- Side effects questionnaire

**Supplementary Table S1.** Cut-off values for different SARS-CoV-2 IgG antibodies and antibodies against four common cold coronaviruses.

| **Target** | **Cut-off (MFI)** |
| --- | --- |
| SARS-CoV-2 Spike | 6800 |
| SARS-CoV-2 Spike S1 | 2700 |
| SARS-CoV-2 Spike RBD | 5800 |
| SARS-CoV-2 Spike S2 | 3200 |
| HCoV-229E Spike S1 | 8012 |
| HCoV-HKU1 Spike S1 | 4235 |
| HCoV-NL63 Spike S1 | 4407 |
| HCoV-OC43 Spike S1 | 3599 |

MFI, mean fluorescence intensity; RBD, receptor-binding domain

**Supplementary Figure S1.** Humoral response in hemodialysis patients before and after a third BNT162b2 vaccine dose with respect to current immunosuppressive therapy.


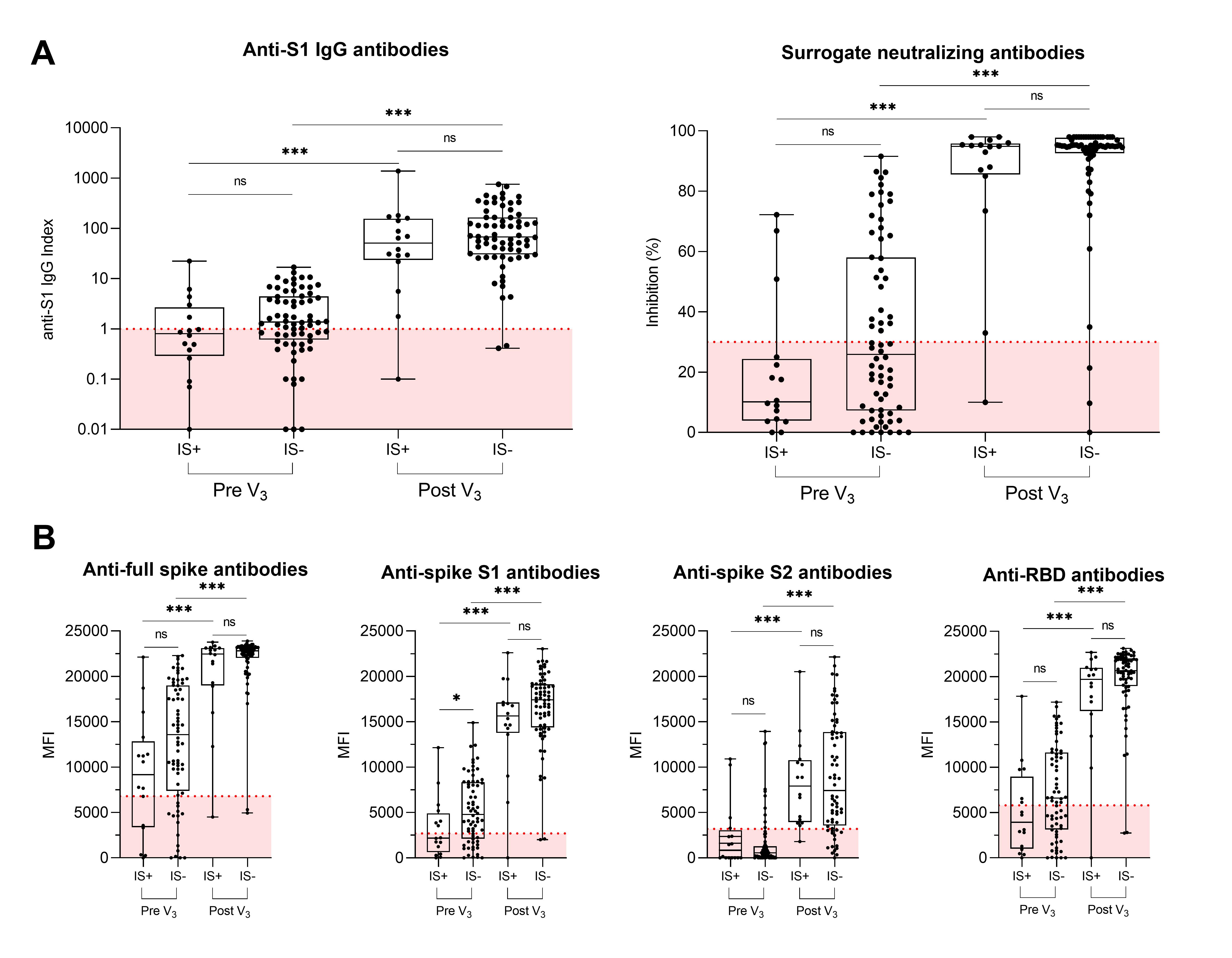


(A) Anti-S1 IgG and surrogate neutralizing antibodies in 84 hemodialysis patients before and after a third BNT162b2 vaccine dose with respect to current immunosuppressive maintenance therapy. An anti-S1 IgG index ≥1 and an inhibition ≥30% as indicated by the dashed red line defined seroconversion for anti-S1 IgG and surrogate neutralizing antibodies, respectively. (B) IgG antibodies against SARS-CoV-2 full spike, spike S1, spike S2 and receptor-binding domain (RBD) as determined by a bead-based multiplex assay. The y-axis represents the mean fluorescence intensity (MFI) with a red dashed line indicating the cut-off for each respective target. Anti-RBD antibodies were chosen to define the seroconverted cohort for later live virus neutralization as the RBD of the SARS-CoV-2 spike protein is a major target of neutralizing antibodies that block viral attachment to the host cell. IS, immunosuppression; MFI, mean fluorescence intensity; RBD, receptor-binding domain; V, vaccination; *** *P*<0.001; ns, non-significant

## Supplementary Figure S2. IgG antibodies against four common cold coronaviruses in 84 hemodialysis patients before and after third BNT162b2 vaccination.


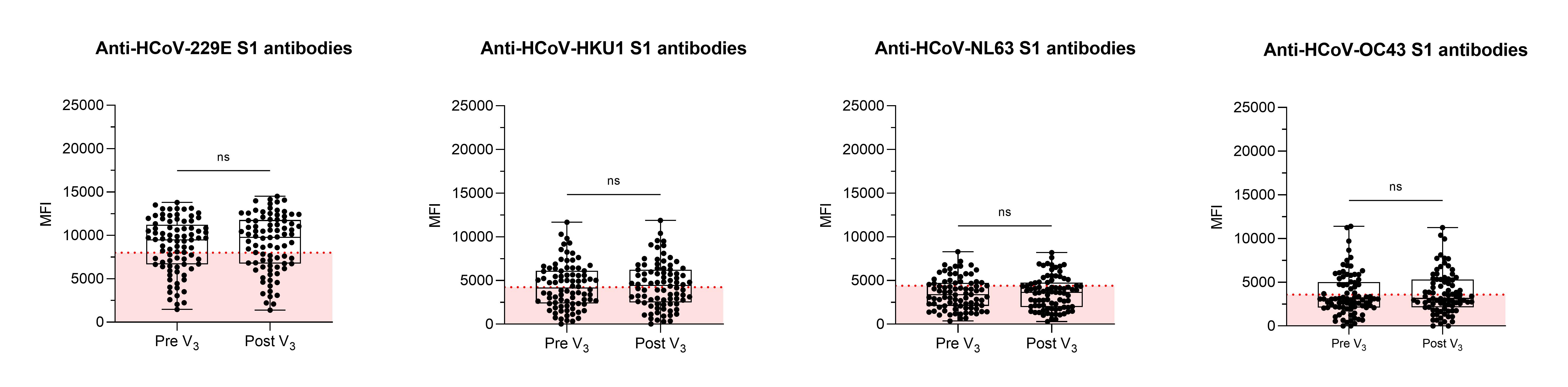


IgG antibodies against the spike S1 of four common cold coronaviruses in 84 hemodialysis patients before and after third vaccination. The y-axis represents the mean fluorescence intensity (MFI) where the dashed red line indicates the cut-off for each target, respectively. MFI, mean fluorescence intensity; ns, non-significant

## Supplementary Figure S3. Individual courses of humoral response in 31 seroconverted hemodialysis patients before and after third BNT162b2 vaccination.


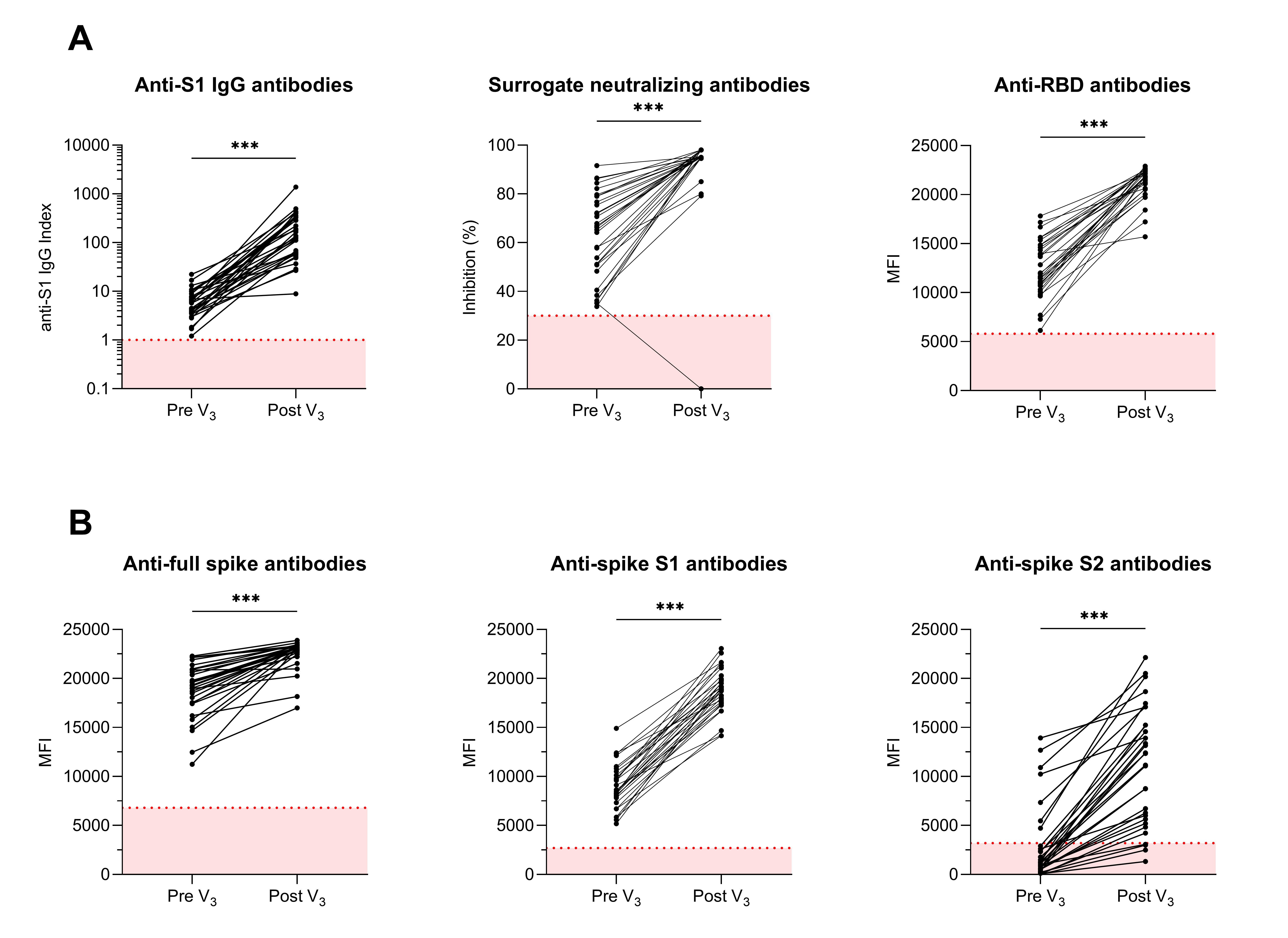


(A) Paired course of anti-S1 IgG antibodies, surrogate neutralizing antibodies and anti-RBD antibodies in 31 hemodialysis patients before and after third vaccination as determined by a chemiluminescent immunoassay, a surrogate virus neutralization test and a bead-based multiplex assay. An index ≥1, an inhibition ≥30% and a mean fluorescence intensity (MFI) ≥3800 defined positivity for each test, respectively. The corresponding cut-offs are indicated by a dashed red line. (B) IgG antibodies against the full spike, the spike S1 and the spike S2 in 31 hemodialysis patients before and after third vaccination as determined by a bead-based multiplex assay. The MFI is given on the y-axis and the respective cut-off for each target is indicated by a dashed red line. RBD, receptor-binding domain; MFI, mean fluorescence intensity; V, vaccination; *** *P*<0.001

**Supplementary Figure S4.** Radial graph for reactogenicity in hemodialysis patients after first, second and third BNT162b2 vaccine dose.


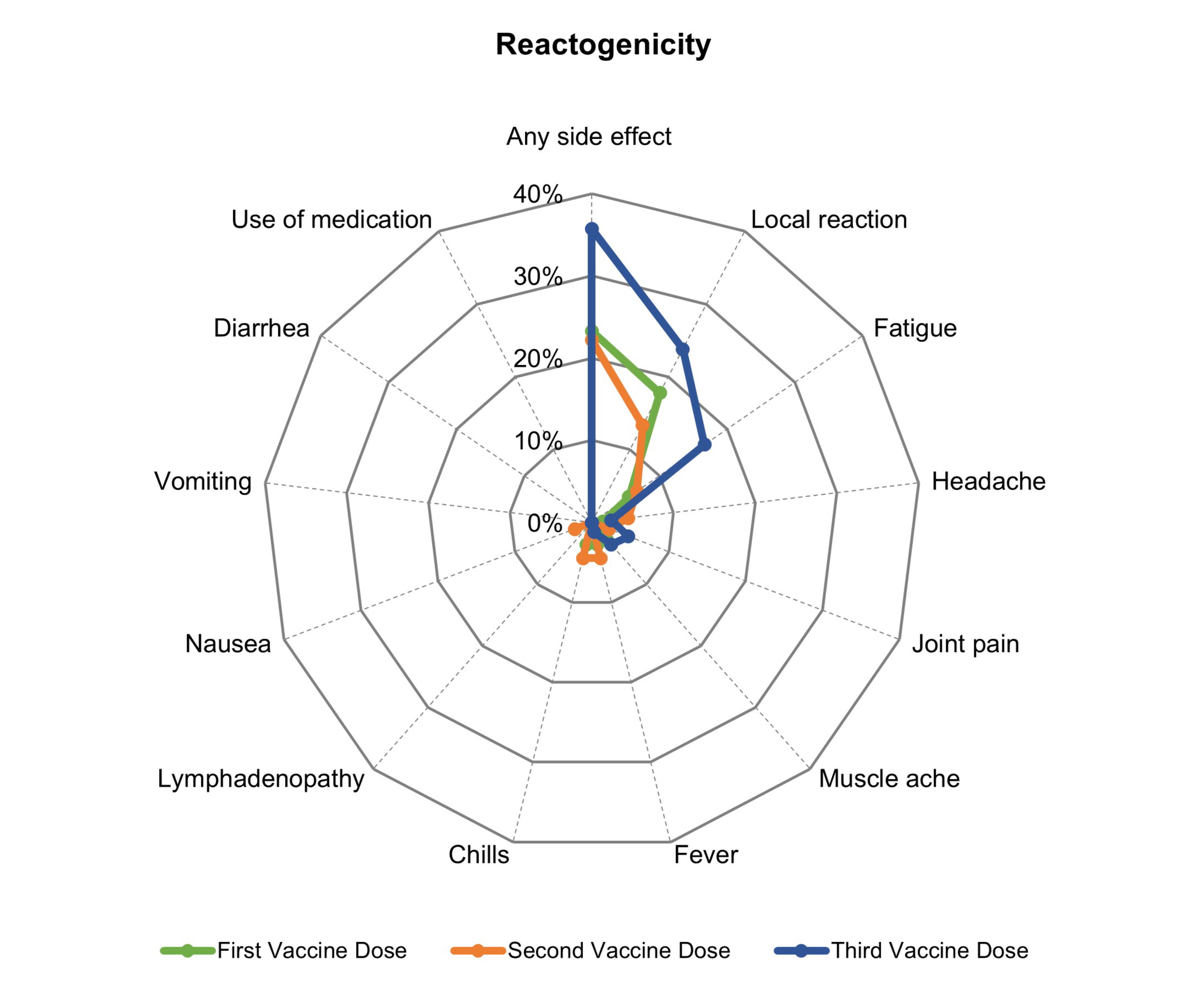


Reactogenicity was assessed after first, second and third BNT162b2 vaccination in 73, 45, and 84 hemodialysis patients, respectively. A 12-item questionnaire was used to inquire common side-effects after COVID-19 vaccination and the use of medication after vaccine reception.

**Supplementary Methods**

Side effects questionnaire

| **Side effects questionnaire** | | | |
| --- | --- | --- | --- |
|  | After first vaccination | After second vaccination | After third vaccination |
| **Any** (yes/no) |  |  |  |
| If **yes**, please mark the appropriate side effects you had: | | | |
|  | After first vaccination | After second vaccination | After third vaccination |
| Local reaction (such as pain at the injection site, redness, swelling) |  |  |  |
| Fatigue |  |  |  |
| Headache |  |  |  |
| Joint pain |  |  |  |
| Muscle ache |  |  |  |
| Fever (≥38°C) |  |  |  |
| Chills |  |  |  |
| Lymphadenopathy |  |  |  |
| Nausea |  |  |  |
| Vomiting |  |  |  |
| Diarrhea |  |  |  |
| Use of medication |  |  |  |
